# An initial clinical evaluation of quantitative susceptibility mapping for quantitative characterization of intramyocardial hemorrhage

**DOI:** 10.1016/j.jocmr.2026.102707

**Published:** 2026-03-03

**Authors:** Andrew Tyler, Matthew E. Li Kam Wa, Simon J. Littlewood, Li Huang, Filippo Bosio, Amedeo Chiribiri, Giulia Benedetti, Sébastien Roujol, Pier Giorgio Masci

**Affiliations:** a School of Biomedical Engineering and Imaging Sciences, King’s College London, St Thomas’ Hospital, London, United Kingdom; b Division of Psychology Communication and Human Neuroscience, University of Manchester, Dover St. Building, Dover Street, Manchester, United Kingdom; c School of Cardiovascular and Metabolic Medicine & Sciences, King’s College London, St Thomas’ Hospital, London, United Kingdom; d Neoscan Solutions GmbH, Magdeburg, Germany; e Radiology Department, Guy’s and St Thomas’ NHS Foundation Trust, London, United Kingdom

**Keywords:** STEMI, IMH, QSM, Iron, Reperfusion injury

## Abstract

**Background:**

Iron deposition following intramyocardial hemorrhage in ST elevation myocardial infarction (STEMI) has a mechanistic role in adverse left ventricular remodeling. Current cardiovascular magnetic resonance imaging-based methods for measuring myocardial iron in STEMI, including T_2_*, T_1_, and T_2_ mapping, are limited. Iron-mediated T_1_, T_2_, and T_2_* relaxation time shortening is opposed by edema-mediated prolongation of relaxation times, hindering the accuracy of these techniques. In contrast, quantitative susceptibility mapping (QSM) measures intrinsic tissue magnetic susceptibility, potentially resulting in higher specificity for iron detection in the setting of infarct-related myocardial edema. The objective of this work is to characterize the performance of QSM for the detection of hemorrhagic iron in the context of STEMI.

**Methods:**

22 patients with STEMI were scanned using QSM and T_2_* mapping sequences. Presence/absence of iron, image quality, and diagnostic confidence were assessed by two expert readers for QSM, T_2_* mapping, and T_2_* weighted imaging (longest echo-time T_2_* map source image). The volume of intramyocardial hemorrhage was quantified for each technique and compared to the volume of microvascular obstruction determined by late-gadolinium enhancement imaging. Mass of hemorrhagic iron in each case was determined using QSM and T_2_* maps.

**Results:**

In the qualitative analysis, QSM had significantly improved diagnostic confidence and image quality compared to both T_2_* maps and T_2_* weighted images. For quantitative analysis, the volume of intramyocardial hemorrhage determined by QSM had a significantly stronger correlation vs the reference standard than both T_2_* map and T_2_* weighted imaging. There was a strong correlation between the mass of hemorrhagic iron calculated by T_2_* map and QSM.

**Conclusion:**

This work demonstrates, in a patient population, the opportunity QSM presents for improving the assessment of hemorrhagic iron in the context of STEMI. Full evaluation in a large clinical trial is now warranted.

## Graphical abstract

**Figure:**
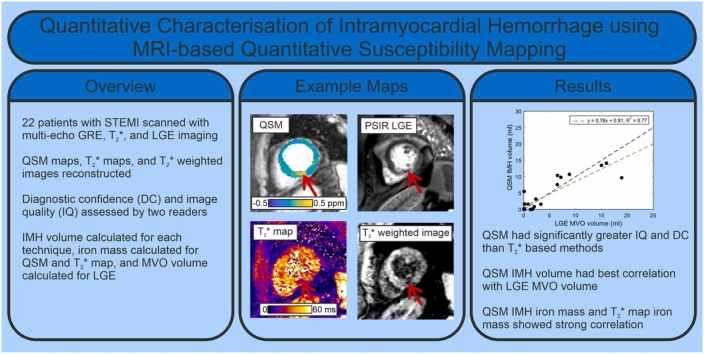

## 1 Background

Iron deposition following intramyocardial hemorrhage (IMH) in ST elevation myocardial infarction (STEMI) has a mechanistic role in adverse left ventricular remodeling [1] and can persist for at least a decade [2]. However, current cardiovascular magnetic resonance (CMR)-based methods for measuring myocardial iron in STEMI, including T_2_*, T_1_, and T_2_ mapping, are limited [3]. Iron-mediated T_1_, T_2_, and T_2_* relaxation time shortening is opposed by edema-mediated prolongation of relaxation times, hindering the diagnostic accuracy of these mapping techniques. In contrast, quantitative susceptibility mapping (QSM) measures intrinsic tissue magnetic susceptibility, potentially resulting in higher specificity for iron detection in the setting of infarct-related myocardial edema. Previously, we reported the repeatability of cardiac QSM in healthy volunteers and example patient cases [4], suggesting potential for clinical utility in patients with suspected IMH.

Comprehensive in vivo evaluation of QSM, in a patient population, for quantification of IMH is challenging, due to the lack of a gold standard method for measuring cardiac iron without post-mortem histology. Despite this, Moon et al. demonstrated good correspondence of QSM signal to tissue iron in a *porcine* model [3]; however, quantitative in vivo validation will be critical to the translation of QSM to the clinic.

In this work, we address this limitation by noting that IMH has close anatomical correlation with microvascular obstruction (MVO) on late gadolinium enhancement (LGE) images (intraclass correlation coefficient = 0.87 in one study [5]), allowing MVO (presence/absence and volume) to be used as an imperfect surrogate for the presence and volume of IMH. It is important to note that MVO without IMH is observed in the literature [6], and caution should therefore be exercised when using MVO as a proxy for IMH. However, the presence/absence of IMH in patients with MVO determined by cardiovascular magnetic resonance (CMR) will depend on the thresholding method used in segmentation, leaving the question of whether true MVO exists (or not) without IMH open [7]. Based on this observation, we report our initial clinical findings using QSM for IMH quantification in patients after STEMI.

## 2 Methods

Thirty-five patients with acute STEMI were recruited to the study; of those, 22 patients were included in analysis (18M/4F, body mass index = 31 ± 5 kg/m^2^, age = 54 ± 12 years, 4.0 ± 2.2 days post-presentation). All patients underwent clinically indicated CMR at 1.5T (MAGNETOM Sola/Aera, Siemens Healthineers, Erlangen, Germany), including phase-sensitive inversion recovery (PSIR) LGE imaging, T_2_ mapping, and long-/short-axis cine imaging. In addition, they received imaging with pre-contrast QSM and T_2_* mapping sequences. Of the participants excluded from the study, 12 did not receive all of LGE, whole heart T_2_*, and QSM imaging, due to the 15-minute acquisition time limitation of our ethical approval, and one was excluded due to incorrect scan setup. Our mean QSM acquisition time was 6.8 ± 1.9 min. QSM and T_2_* mapping were acquired in the same orientation as the cine short-axis stack covering the whole left ventricle. The QSM acquisition consisted of a multi-echo three-dimensional-gradient-recalled echo (GRE) research sequence (five echoes, TE_1_ = 3.2 ms, ∆TE = 2.9 ms, typically field-of-view (FOV) = 288 × 384 × 100 mm^3^ and voxel size = 1.5 × 1.5 × 5 mm^3^) and was reconstructed with the Morphology Enhanced Dipole Inversion toolbox [8] as described in Tyler et al. [4]. The T_2_* map acquisition consisted of an eight-echo two-dimensional-GRE sequence (TE_1_ = 2.1 ms, ∆TE = 2.0 ms, and typically FOV = 325 × 400 mm^2^ with slice thickness = 8 mm and voxel size = 1.6 × 1.6 mm^2^). For T_2_* imaging, IMH was assessed with both the reconstructed maps and T_2_* weighted source images with the longest echo time (16.22 ms).

**Fig. 1:**
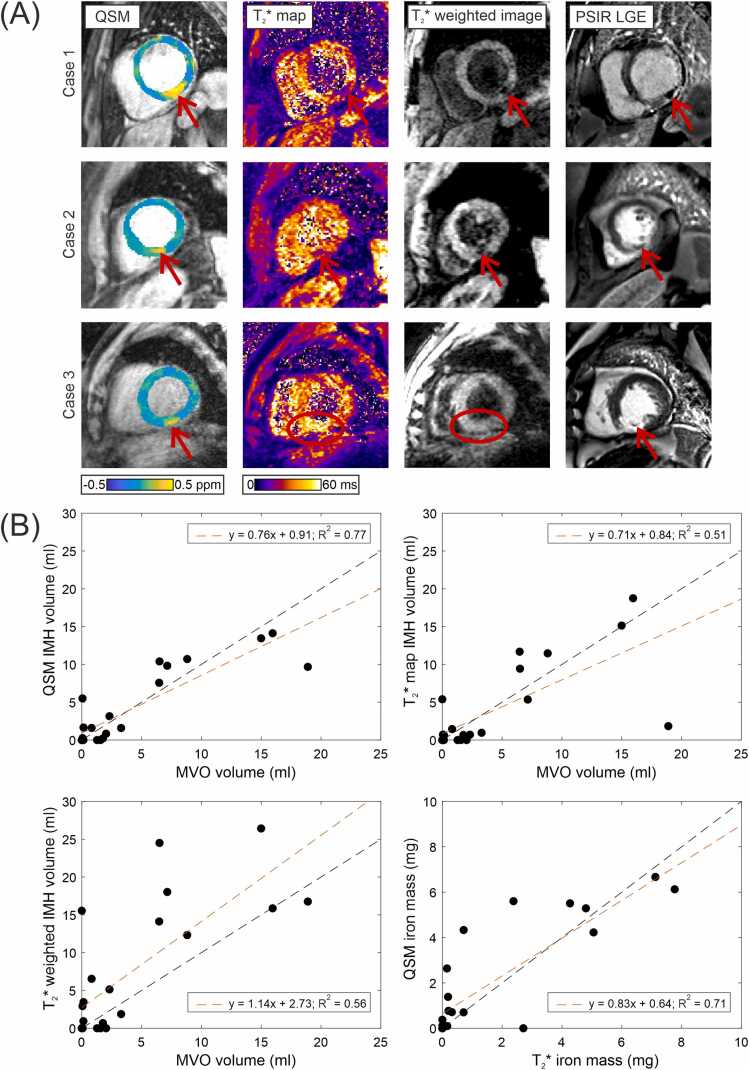
(A) Example images from three cases imaged after STEMI. The IMH is clearly visualized by QSM in all cases. T_2_* map and T_2_* weighted images visualize IMH in cases 1 and 2 only. MVO is visualized in the PSIR LGE images in all cases. (B) Summary of quantitative results. Correlation of IMH volume quantified with each technique against MVO volume and correlation of iron quantified with QSM and T_2_* map techniques. Raw QSM images are shown in Supplementary Figure 2. *STEMI* ST elevation myocardial infarction, *IMH* intramyocardial hemorrhage, *QSM* quantitative susceptibility mapping, *MVO* microvascular obstruction, *PSIR* phase-sensitive inversion recovery, *LGE* late gadolinium enhancement

For IMH diagnosis (IMH present/absent), all images were qualitatively assessed by the consensus of two expert readers (blinded to patient characteristics), who also assessed the presence/absence of MVO in the LGE images and edema in the T_2_ maps. The images were also scored based on a 5-point Likert scale (very poor, poor, neutral, good, very good, with neutral indicating clinical utility; see Supplementary Fig. 1) for diagnostic confidence (DC) and image quality (IQ) and averaged in the case of disagreement. Both QSM and T_2_* maps were presented to the readers as raw maps and overlay images (Fig. 1A). A paired Wilcoxon signed-rank test was used to compare qualitative scores.

For IMH quantification, MVO volume was quantified on the LGE images (remote − 5 standard deviation [SD] threshold) and compared to the volume of IMH in the T_2_* weighted images, T_2_* map (1/T_2_* is used for thresholding as this is approximately linearly dependent on iron concentration), and QSM images. In each slice, the endothelial and epithelial wall of the left ventricle were segmented, then a remote, non-infarcted region was identified, and the region surrounding the infarct was manually segmented, excluding artifacts. The mean and spatial SD of the remote region were calculated, and the IMH was segmented using a threshold of remote ± 1 − 5 SD (± as appropriate). In each case, the volume reported was calculated by taking the number of segmented voxels and multiplying by the volume of one voxel. For each technique and threshold, the IMH volume was plotted against LGE MVO volume and a linear fit calculated; the threshold with the best R^2^ was then selected. The strength of the correlation of IMH volume with LGE MVO volume for QSM was compared to T_2_*-based techniques with Zou’s method for overlapping correlations [9]. Additionally, the mass of iron deposition was quantified with the T_2_* and QSM maps according to Fe([mg/g], wet weight) = (1/5.04) × 45.0 × (T_2_*[ms])^−1.22^ [10] and Fe([mg/g], wet weight) = 0.343 + 0.625 × Δχ[ppm] [11]. For all comparisons, α = 0.05 was considered significant.

## 3 Results

Example cases are shown in Fig. 1A. In the qualitative analysis, QSM had significantly higher DC and IQ than T_2_* weighted images and T_2_* maps (QSM DC = 4.6 ± 0.6, T_2_*map DC = 4.0 ± 0.9 (p = 0.008), T_2_* weighted DC = 3.8 ± 1.0 (p = 0.002), QSM IQ = 4.4 ± 0.6, T_2_*map IQ = 3.9 ± 0.7 (p = 0.002), T_2_* weighted IQ = 3.6 ± 1.0 (p = 0.002)). Images were rated as “good” (score ≥4) for DC in 91% (20/22) of QSM, vs 64% (14/22) of T_2_*map and 64% (14/22) of T_2_* weighted images. For IQ, this was 82% (18/22) for QSM vs 64% (14/22) of T_2_*map and 50% (11/22) of T_2_* weighted images. Expert assessment of presence/absence of IMH (Table 1) returned 18 concordant pairs between QSM and T_2_* based imaging (T_2_* map and T_2_* weighted images returned the same results). Of the four discordant pairs, three were plausible based on the presence/absence of MVO; however, T_2_* imaging returned IMH+ in the absence of MVO in one case. All cases were positive for edema in the infarct region on T_2_ mapping.

**Table 1:** Truth table comparing IMH diagnosis for QSM and T_2_* imaging.^b^

	T_2_* IMH+	T_2_* IMH−
QSM IMH+	13 (13)	2 (2)
QSM IMH−	2 (1)	5 (2)

*IMH* intramyocardial hemorrhage, *QSM* quantitative susceptibility mapping, *MVO* microvascular obstruction

b Number of cases for each combination of QSM IMH +/- and T_2_* IMH +/-. The number of MVO positive cases for each category is shown in brackets. Note that T_2_* map and T_2_* weighted images returned the same results in all cases.

For quantitative analysis (Fig. 1B), the thresholds for each technique with the greatest correlation to IMH volume determined LGE imaging were QSM = remote + 2 SD (R = 0.875, 95% confidence interval [CI] = [0.7184, 0.9472]), T_2_* weighted image = remote − 1 SD (R = 0.748, 95% CI = [0.4760, 0.8890]), 1/T_2_* map = remote + 4 SD (R = 0.716, 95% CI = [0.4219, 0.8739]). Using Zou’s method, R(QSM) − R(T_2_* map) had 95% CIs (0.0344, 0.4090) and R(QSM) − R(T_2_* weighted image) had CIs (0.0022, 0.3585), indicating that the IMH volume for QSM had a significantly stronger correlation with LGE MVO volume than either T_2_* map or T_2_* weighted images. There was a strong correlation between iron mass in the IMH calculated by T_2_*map and QSM (R = 0.8404).

## 4 Discussion

Overall, both the quantitative and qualitative results suggest that QSM had superior diagnostic performance at 1.5T than both T_2_* mapping and T_2_* weighted imaging for the detection of IMH. The expert readers preferred QSM to T_2_* based methods, with significantly improved DC and IQ. While overall IQ was improved for QSM vs T_2_* based imaging, QSM (and T_2_*) were in some cases compromised by susceptibility artifacts, which could potentially obscure true IMH. QSM IMH volume also had significantly stronger correlation with MVO volume compared to T_2_*-based methods, potentially indicating improved robustness to artifacts. This conclusion is supported by Moon et al. [3], who qualitatively observed that QSM had improved robustness to edema and fibrosis than T_2_*-based techniques in a smaller cohort.

Furthermore, the strong correlation between hemorrhagic iron mass measured with QSM and T_2_* mapping suggests that QSM could be used not only for risk stratification of patients with STEMI, but also for quantitative assessment of iron chelating treatments to remove cardiac iron in IMH.

Beyond hemorrhagic iron quantification, the good correlation of QSM with MVO volume may present an opportunity for comprehensive non-contrast assessment of MI when combined with T_1_ mapping. This would both eliminate the dependence of scar and MVO quantification on contrast kinetics and, with inline reconstruction of QSM (which would require a reduction in reconstruction time from ∼5 min currently), enable a significant reduction in CMR exam times.

The principal limitations of this study are the cohort size of 22 patients, single-center design, and lack of long-term follow-up data, all of which we hope to be able to address in future large-scale studies.

## 5 Conclusion

QSM presents a significant opportunity for improving the assessment of IMH in patients. IMH volume measured with QSM had improved correlation to MVO volume compared to T_2_*-based techniques and was preferred by two expert readers. Evaluation in a large clinical trial is now warranted.

## Funding

10.13039/501100000274British Heart Foundation (BHF), Grant/Award Numbers: (PG/19/11/34243), (PG/21/10539); 10.13039/501100000266Engineering and Physical Sciences Research Council (EPSRC), Grant/Award Number: (EP/R010935/1); Biomedical Research Centre at Guy's and St Thomas' National Health Service Foundation Trust (NHS); 10.13039/501100000764King's College London; this work represents independent research supported by the National Institute for Health and Care Research (NIHR) Clinical Research Facility (CRF) and the NIHR HealthTech Research Centre (HRC) in Cardiovascular and Respiratory Medicine at Guy’s and St Thomas’ NHS Foundation Trust and King’s College London. The views expressed are those of the authors and not necessarily those of the NHS, the NIHR, or the Department of Health and Social Care. This research was funded in whole, or in part, by the 10.13039/100004440Wellcome Trust (WT 203148/Z/16/Z). For the purpose of open access, the author has applied a CC BY public copyright licence to any Author Accepted Manuscript version arising from this submission.

## Author contributions

**Matthew E. Li Kam Wa:** Writing – review & editing, Investigation. **Simon J. Littlewood:** Writing – review & editing, Investigation. **Li Huang:** Writing – review & editing, Software. **Filippo Bosio:** Writing – review & editing, Resources, Project administration. **Andrew Tyler:** Writing – original draft, Methodology, Investigation, Formal analysis, Conceptualization. **Amedeo Chiribiri:** Writing – review & editing, Resources, Project administration, Funding acquisition. **Giulia Benedetti:** Writing – review & editing, Methodology, Formal analysis. **Sébastien Roujol:** Writing – review & editing, Supervision, Methodology, Funding acquisition, Conceptualization. **Pier Giorgio Masci:** Writing – review & editing, Methodology, Investigation, Formal analysis, Conceptualization.

## Ethics approval and consent

All healthy volunteers and patient participants gave informed written consent for this study, which had research ethics committee approval (approval numbers 15/NS/0030 and 23/NS/0034), and was in full compliance with the Declaration of Helsinki.

## Consent for publication

Consent for publication was obtained from all participants in the study.

## Declaration of competing interests

The authors declare the following financial interests/personal relationships which may be considered as potential competing interests: Sébastien Roujol reports financial support was provided by the British Heart Foundation. Sébastien Roujol reports financial support was provided by the Engineering and Physical Sciences Research Council. All Authors reports financial support was provided by NIHR Biomedical Research Centre at Guy’s and St Thomas’ NHS Foundation Trust and King’s College London. All authors report financial support was provided by NIHR Clinical Research Facility at Guy’s and St Thomas’ NHS Foundation Trust and King’s College London. All authors report financial support was provided by NIHR HealthTech Research Centre at Guy’s and St Thomas’ NHS Foundation Trust and King’s College London. All authors report financial support was provided by Wellcome Trust. Pier Giorgio Masci reports a relationship with Perspectum Ltd. that includes consulting or advisory. Andrew Tyler and Sébastien Roujol have a patent pending to Siemens Healthineers. Sébastien Roujol has patent with royalties paid to Siemens Healthineers. The other authors declare that they have no known competing financial interests or personal relationships that could have appeared to influence the work reported in this paper.

## Data Availability

The datasets used and/or analyzed during the current study are available from the corresponding author on reasonable request.
