# Supplementary material for: An initial clinical evaluation of quantitative susceptibility mapping for quantitative characterization of intramyocardial hemorrhage
Source: J Cardiovasc Magn Reson. 2026 Mar 3;28(1):102707. doi: 10.1016/j.jocmr.2026.102707 (PMC13208779; doi:10.1016/j.jocmr.2026.102707)

An Initial Clinical Evaluation of Quantitative Susceptibility Mapping for Quantitative Characterisation of Intramyocardial Hemorrhage: Supplementary Information

**Supplementary Figure 1:** Three example cases with expert reader scores for image quality (IQ) and diagnostic confidence (DC). Red arrow indicated identified IMH. Note that in the case of disagreement between the two expert readers the mean score was taken.

**
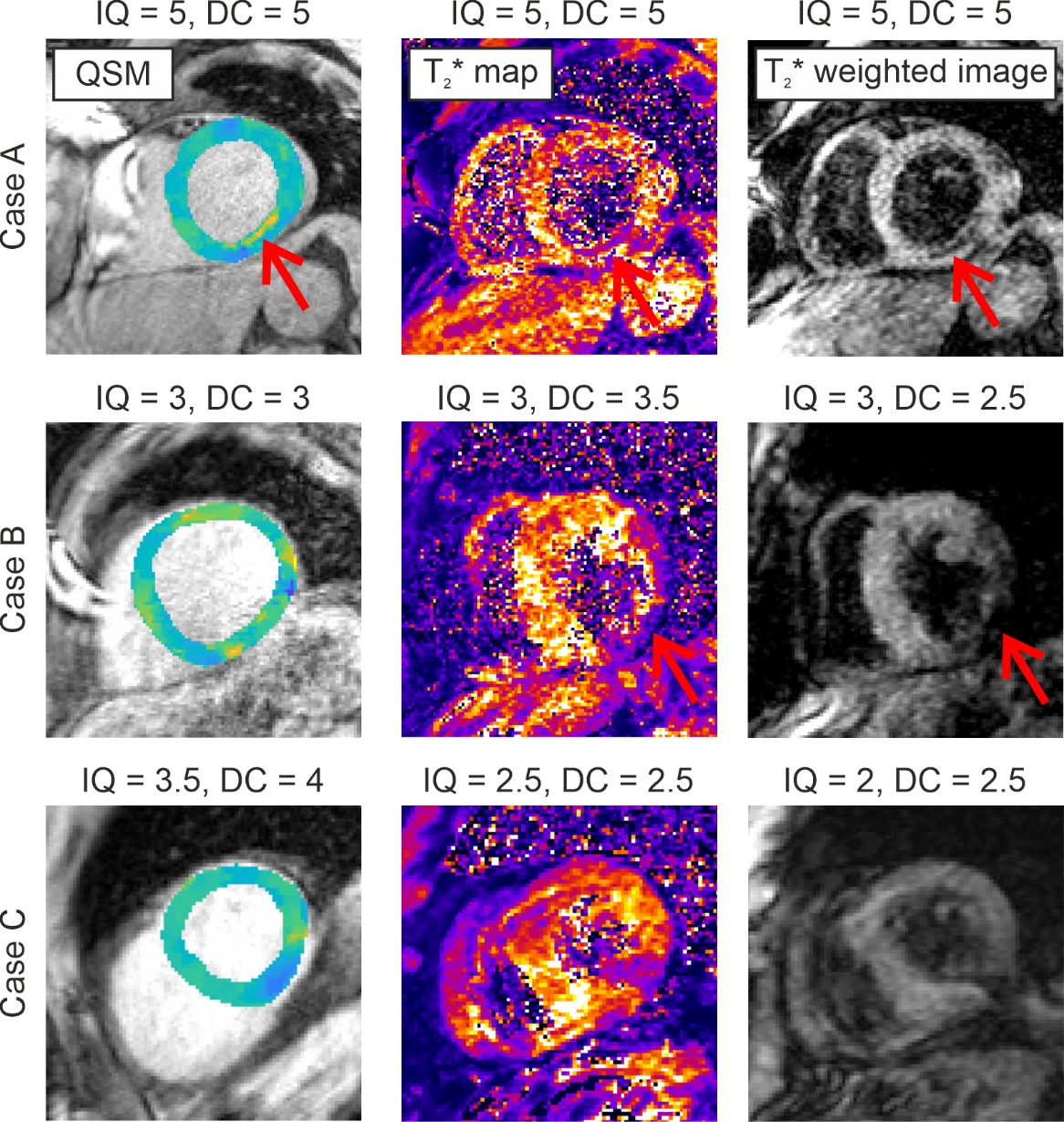
**

**Supplementary Figure 2:** Expanded version of Figure 1 with additional raw QSM images and T_2_ maps.


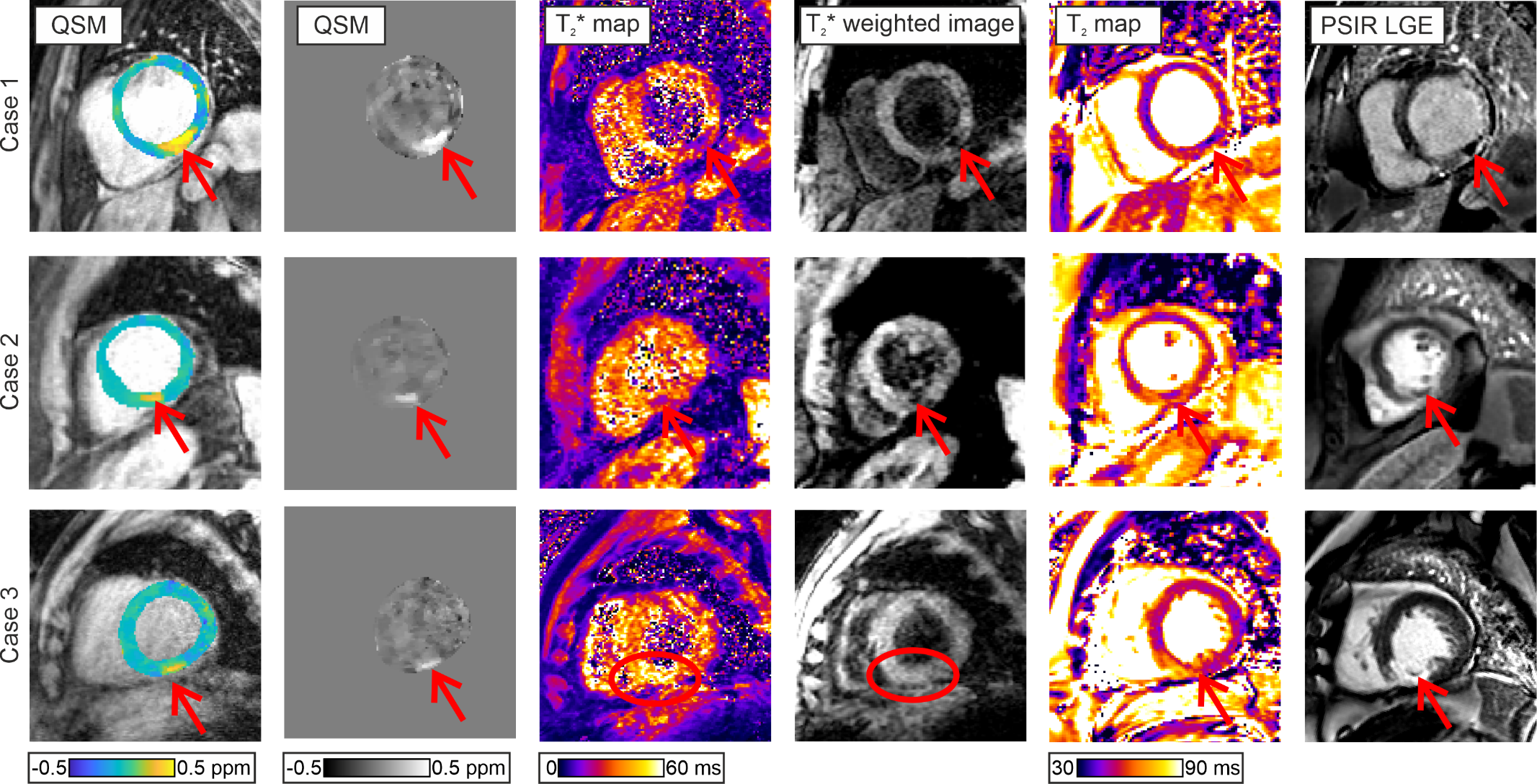

Supplement: Supplementary file 1 — Supplementary material [file mmc1.docx]
